# Optimizing lower intensity triplet therapy in acute myeloid leukemia: a practical guide

**DOI:** 10.1038/s41408-025-01429-z

**Published:** 2025-11-25

**Authors:** Wei-Ying Jen, Curtis A. Lachowiez, Jennifer Marvin-Peek, Jessica K. Altman, Musa Yilmaz, Jacqueline S. Garcia, Yasmin Abaza, Nicholas J. Short, Joshua F. Zeidner, Naval G. Daver, Andrew H. Wei, Ghayas C. Issa, Courtney D. DiNardo

**Affiliations:** 1 Department of Leukemia, The University of Texas MD Anderson Cancer Center, Houston, TX USA; 2 Division of Hematology/Oncology, Oregon Health & Science University, Portland, OR USA; 3 Division of Hematology/Oncology, Department of Medicine, Robert H. Lurie Comprehensive Cancer Center, Northwestern University, Chicago, IL USA; 4 Division of Leukemia, Dana-Farber Cancer Institute, Boston, MA USA; 5 Division of Hematology, Department of Medicine, Lineberger Comprehensive Cancer Center, University of North Carolina, Chapel Hill, NC USA; 6 Peter MacCallum Cancer Centre, Royal Melbourne Hospital and Walter and Eliza Hall Institute of Medical Research, Melbourne, VIC Australia

**Keywords:** Combination drug therapy, Molecularly targeted therapy

## Abstract

Venetoclax-based doublets with azacitidine or low dose cytarabine are the standard of care for the treatment of acute myeloid leukemia (AML) in older patients or those unfit for intensive chemotherapy. However, some patients do not attain complete remission, and over time, most patients relapse. Frontline triplet therapy incorporating a targeted therapy (FLT3, IDH or menin inhibitor) is an emerging treatment concept under investigation for this population. Initial triplet regimens have yielded encouraging composite complete remission and measurable residual disease negativity rates, enabling the transition to allogeneic stem cell transplantation for eligible patients. While effective, triplets are associated with myelosuppression and cytopenia-related toxicities, which can affect treatment tolerability and quality of life. In this review, we summarize the available evidence for triplet therapy in AML and offer our recommendations on the practical application of triplets in clinical practice, with particular focus on adjustments to dosing schedules in induction and continuation cycles. We also outline drug-specific adverse effects and interactions based on emerging clinical data to help guide the clinician, given the increasing use of novel combination therapies.

## Introduction

Acute myeloid leukemia (AML) is a hematological malignancy characterized by clonal diversity and evolution [1–3]. Prior to the approval of venetoclax (VEN), the treatment options for patients unfit for intensive chemotherapy (IC) included monotherapy with hypomethylating agents (HMA, azacitidine [AZA] or decitabine [DAC]), or low-dose cytarabine (LDAC) [4]. The incorporation of VEN into frontline regimens with AZA or LDAC has revolutionized the treatment of older adults with AML, increasing composite complete remission (CRc) rates to two-thirds of patients and median overall survival (OS) to 14.7 months [5, 6]. However, longer follow-up has also established that responses with VEN doublets are generally not durable, with a 2-year OS of 20–40% [7, 8]. Real-world studies have subsequently reported less impressive outcomes, with median OS ranging from only 9 to 12 months [9]. Despite improved tolerability compared to standard IC regimens, protracted myelosuppression and cytopenia-related infections are common with VEN combinations. Increased cycle length (often 5–6 weeks) with reduced VEN durations (typically 7–21 days per cycle) along with growth factor support are now frequently recommended in continuation cycles to improve tolerability of VEN doublets [9, 10].

**Fig. 1:**
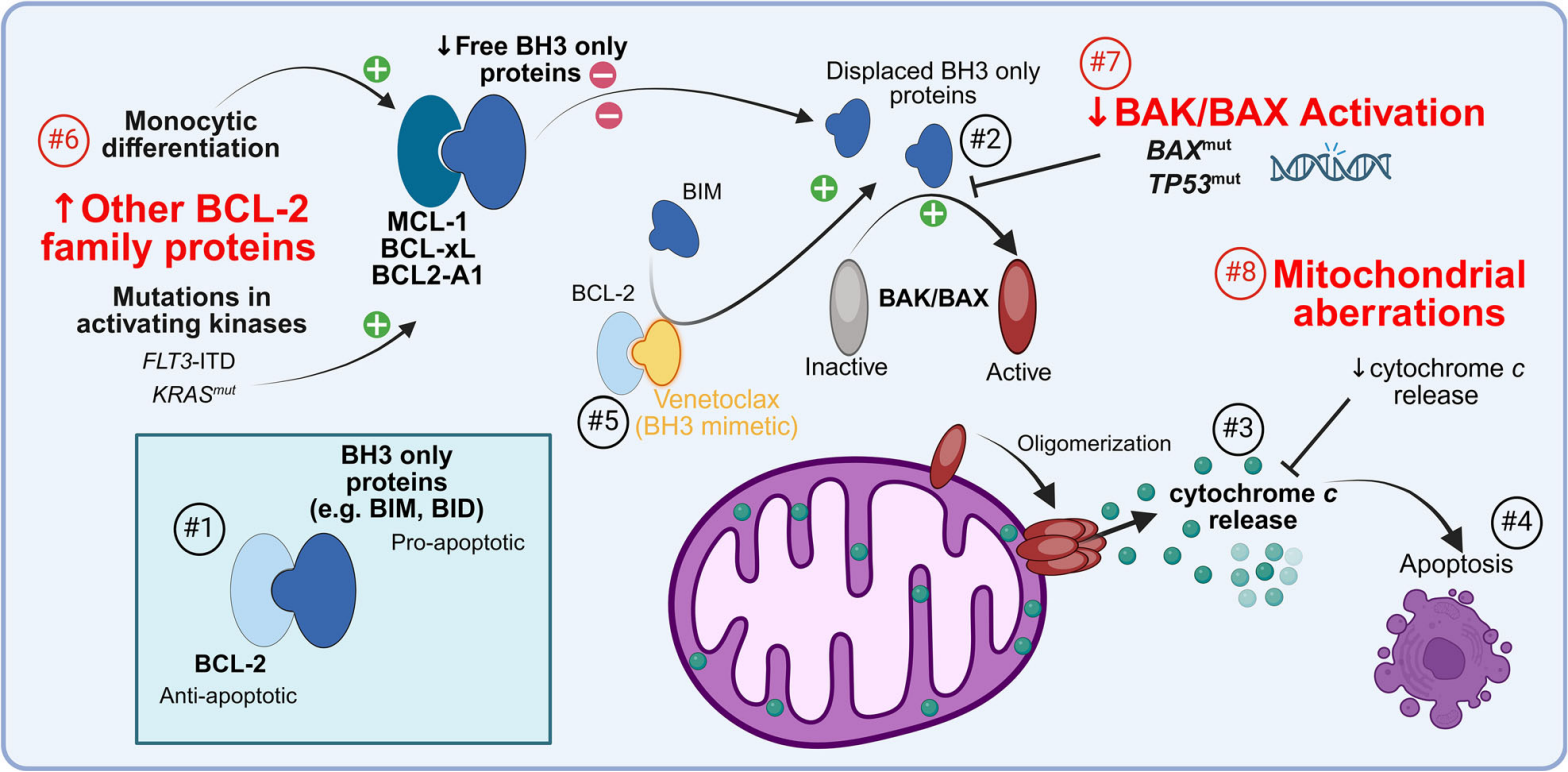
Mechanisms of resistance to venetoclax. In the normal apoptotic pathway, the pro-apoptotic BH3 only proteins are bound to BCL-2 (#1). Apoptosis is triggered by displaced BH3 proteins, which activate BAK/BAX (#2) and release cytochrome *c* from the mitochondria (#3-4). Venetoclax binds to BCL-2 (#5), causing displacement of the BH3 protein BIM and initiating apoptosis through the BAK/BAX activation pathway. Resistance to venetoclax can develop by an increase in other BCL-2 family proteins such as MCL-1 and BCL-xL (#6), which bind to free pro-apoptotic BH3 proteins and decrease BAK/BAX activation. Alternatively, *BAX* or *TP53* mutations can prevent activation of BAK/BAX, preventing cytochrome c release and decreasing apoptosis through this pathway (#7). Finally, mitochondrial aberrations that result in decreased cytochrome c release are also resistant to venetoclax-mediated apoptosis (#8).

Relapse and resistance to VEN therapy are multifactorial. VEN is a BH3 mimetic which binds to the anti-apoptotic protein BCL2, disrupting the interaction between BCL2 and BH3-only proteins such as BIM, leaving them free to bind apoptotic effectors BAX and BAK. Oligomerization of BAX/BAK effectors permeates the outer mitochondrial membrane, resulting in cytochrome c release and activation of caspase-dependent cell death [11, 12]. Mechanisms of resistance to VEN may target any step in this pathway, including: (i) activation of RAS/MAPK pathway and enhanced expression of pro-apoptotic proteins (e.g., BCL2, BCL-xL or MCL-1) [13, 14]; (ii) reduced expression or activation of apoptotic effectors (BAX and BAK are targets of TP53 and levels are reduced in *TP53* knockout models) [15]; and/or (iii) mitochondrial aberrations which reduce cytochrome c release (Fig. 1) [16]. Concerns about response durability with VEN doublets, and preclinical data suggesting synergism between targeted agents and VEN have led to the development and investigation of various triplet regimens in AML [17–19].

In this review, we outline the evidence supporting the use of lower intensity triplet regimens in AML, with a focus on the newly diagnosed (ND) AML setting. We acknowledge that most of the data discussed are from phase 2 trials, with phase 3 confirmatory trials ongoing. We highlight practical prescribing considerations vis-à-vis agent selection and suggested modifications, both in terms of dose adjustments and changes to duration of therapy, to minimize myelosuppression while preserving efficacy.

## General considerations

Triplet therapy may be considered for patients who are ineligible for intensive chemotherapy. Although there was no upper age limit defined in most of the established triplet studies, careful patient selection is required for patients aged ≥75 due to the myelosuppression associated with triplets.

Throughout this paper, we suggest dose adjustments to minimize myelosuppression. A few general dosing considerations are important and apply to all situations where VEN is used. For example, the interactions of VEN with CYP3A inhibitors are well known [5]. Doses of VEN should be reduced by 50% in the presence of moderate CYP3A inhibitors (e.g., isavuconazole, fluconazole) and at least 75% with strong CYP3A inhibitors (e.g., voriconazole). Concurrent administration with posaconazole specifically requires further dose reductions from 400 mg to 50 mg or 70 mg daily [20].

Induction therapy for ND AML with triplet regimens should generally take place in the inpatient setting, with transition to outpatient for continuation cycles. As with VEN doublets, cytoreduction of circulating white blood cells to <25 × 10^9^/L prior to initiating therapy is advised to mitigate the risk of tumor lysis syndrome. Supportive care with antimicrobial prophylaxis in accordance with local guidance, use of granulocyte colony-stimulating factor (GCSF) in remission, and close monitoring of blood counts is essential. In the first outpatient cycle, we recommend patients be monitored three times per week. The frequency of this may be reduced when a tolerable dose schedule is achieved, and transfusion requirements are predictable.

Although suggested doses and schedules are presented below, therapy should be tailored to individual patients, and further reductions may be necessary if myelosuppression in subsequent continuation cycles remains protracted (>42 days).

## FLT3 Inhibitors

Fms-like receptor tyrosine kinase 3 (FLT3) is a transmembrane tyrosine kinase receptor which mediates signaling and cell survival pathways when activated by its extracellular FLT3 ligand. The most common *FLT3* mutations (*FLT3*^mut^) are internal tandem duplications (ITD), followed by those in the tyrosine kinase domain (TKD). *FLT3*^mut^ AML typically presents with proliferative disease, and *FLT3*-ITD is associated with high early relapse rates in the absence of a FLT3 inhibitor (FLT3i) [21]. *FLT3*^mut^ are present in ~30% of ND AML (20% in older adults), and may be acquired in up to 20% of relapsed/refractory (RR) AML [22–25]. The introduction of FLT3i such as midostaurin [26], gilteritinib [27–29], quizartinib [30, 31], sorafenib [32, 33] and crenolanib [34], have significantly improved outcomes of patients with *FLT3*^mut^ AML in both the ND and RR setting, especially in frontline combination with IC for fit, younger patients.

Older patients may not benefit from IC combinations due to early morbidity and mortality. Subgroup analysis from QuANTUM-First showed no survival benefit of quizartinib over placebo with 7 + 3 in patients aged over 60 years [31]. Older patients with *FLT3*-ITD mutations receiving HMA-based regimens also fare poorly, with no survival advantage with either AZA + VEN (median OS 10 months) [35, 36], or AZA and gilteritinib (median OS 10 months) versus AZA alone (median OS 9 months) [37]. Relapses are frequent and characterized by emergence of signaling mutations (i.e., *KRAS*/*NRAS*) or alternate *FLT3*^mut^ [38–42]. Pre-clinical data suggests FLT3i synergism with VEN through FLT3i-mediated downregulation of MCL-1 and BCL-xL [43, 44]. Additionally, retrospective studies have suggested that triplet therapy is associated with significantly higher rates of CRc (93% versus 70%) compared with an HMA + FLT3i doublet [45]. Consequently, several triplet strategies are under investigation in an effort to improve response rates and survival outcomes in *FLT3*^mut^ AML [46].

### FLT3 triplets

**Fig. 2:**
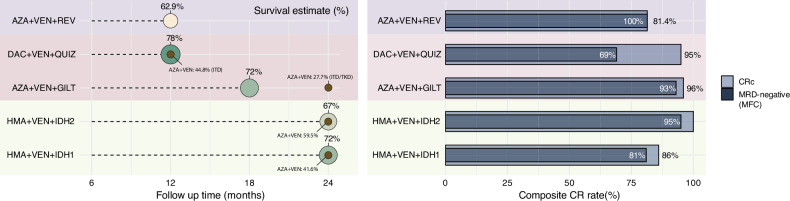
Survival and response rates with triplet combinations. Left panel: OS percentage for different triplet regimens plotted against follow-up duration to reflect that studies report survival at different timepoints. Each regimen is represented by a colored circle; the area of the circle is proportional to the survival percentage, with the numeric survival value is shown adjacent to the circle. Where available, VIALE-A subgroup survival values are illustrated as brown circles to distinguish them from triplet survival rates. Note that differing follow-up intervals should be considered when interpreting survival data. Right panel: response outcomes for the same triplet combinations CRc and MRD undetectable rates (measured by MFC), AZA + VEN + REV: azacitidine, venetoclax and revumenib; DAC + VEN + QUIZ: decitabine, venetoclax and quizartinib; AZA + VEN + GILT: azacitidine, venetoclax and gilteritinib; HMA + VEN + IDH2: hypomethylating agent + venetoclax + enasidenib; HMA:VEN + IDH1: hypomethylating agent + venetoclax + ivosidenib; CRc composite complete remission; MRD measurable residual disease; MFC multiparameter flow cytometry.

A phase 2 study of AZA + VEN with gilteritinib (AZA + VEN + GILT) was recently reported. Thirty patients with ND *FLT3*^mut^ AML and a median age of 71 (73% with *FLT3*-ITD, 27% with *FLT3*-TKD) received AZA + VEN + GILT, with CRc rates of 96% and a median OS which was not reached at median follow-up 19 months (18-month OS 72%, 18-month relapse-free survival 71%, Fig. 2) [47]. The 18-month OS was 61% for *FLT3*-ITD and 100% for *FLT3*-TKD. Amongst patients with at least one *FLT3*-ITD measurable residual disease (MRD) assessment by next-generation sequencing (NGS sensitivity of 5 × 10^−5^) at the end of cycle 2 or later, the MRD negative rate was 76%. As *FLT3*-ITD mutations are independently associated with higher odds of central nervous system (CNS) relapse [48], two doses of intrathecal cytarabine prophylaxis were administered to 26 (87%) patients.

The combination of DAC, VEN and quizartinib (DAC + VEN + QUIZ) was evaluated in a separate phase 2 study specific to patients with *FLT3*-ITD mutations [49]. The median age was 70, with 26% aged ≥75 years. The ND cohort comprised 26 patients, 15 with (58%) de novo and 11 (42%) secondary or therapy-related disease. The CRc rate was 92%, with 67% MRD-negative by multiparameter flow cytometry (MFC). The 30-day mortality was 4%; at a median follow-up of 17 months, the median OS was not reached.

Pooled analysis of the FLT3 triplet experience at The University of Texas MD Anderson Cancer Center (MDACC) in 88 patients (77% *FLT3*-ITD, median age 70) with a median follow-up of 26 months showed a 3-year OS of 46% and 3-year relapse-free survival of 40% [50].

Triplets utilizing an LDAC + VEN backbone have also been explored. The Australian Leukaemia and Lymphoma Group (ALLG) conducted a multi-center, randomized phase 2 trial of patients with AML with intermediate risk cytogenetics. Patients were randomized to a combination of LDAC + VEN with midostaurin (LVM) or LDAC + VEN (INTERVENE, ALLG AMLM25) [51]. Midostaurin was chosen because of its short half-life (19 h versus 113 h for gilteritinib), and was administered sequentially to LDAC to mitigate the risk of myelosuppression. Like DAC + VEN + QUIZ and AZA + VEN + GILT, patients with HMA-failure secondary AML were eligible. The trial enrolled 120 patients in a 2:1 randomization (79 LVM, 41 LDAC + VEN); 29 (22%) were *FLT3*-ITD (22 [28%] in the LVM arm). The median age in the LVM arm was 74, with 27% aged ≥80. In the *FLT3*-ITD sub-group, CRc was obtained in 82% of LVM-treated patients, compared with 57% after LDAC + VEN; 61% cleared *FLT3*-ITD using a highly sensitive NGS assay. Median OS was 16.8 months, with a 1-year OS of 57%. Of interest, 4/9 (44%) of relapses after LVM were negative for *FLT3*-ITD. Importantly, sequential midostaurin did not impair the ability to deliver treatment, with similar median inter-cycle lengths (approximately 35 days) in both arms.

### Recommended triplet doses and schedules

A major concern with FLT3i triplets is myelosuppression, and adjustments to VEN and FLT3i dosing schedules are required to balance efficacy and safety. Initial triplet combinations using doses of 120 mg of gilteritinib in ND patients reported a median of 44 and 34 days to absolute neutrophil count (ANC) > 0.5 × 10^9^/L and platelets >50 × 10^9^/L, respectively [52]. In the BEAT AML trial, a 3 + 3 design to determine the optimal dose of gilteritinib in combination with DAC and VEN used a schedule of 120 mg on days 1–7, followed by decreasing durations of 80 mg for the remainder of the cycle [53]. Hematologic dose-limiting toxicities (DLTs) were noted at the -2 dose level (80 mg D8-14), requiring further dose reduction to 80 mg from day 1. Based on a better balance of safety/efficacy in a dose-escalation study in RR AML, gilteritinib should be dosed at 80 mg in triplet combinations, not the 120 mg monotherapy dose approved by the United States (US) Food and Drug Administration (FDA, Table 1) [47].

**Table 1:** Drugs and suggested doses/dose modifications.

Class	Drug	Single agent dose	LIT triplet dose	Major drug interactions & combination dose adjustments	Specific grade ≥ 3 AEs of clinical concern^a^
**BH3 mimetic (BCL2 inhibitor)**	**Venetoclax**	800 mg daily^b^ [112]	400 mg daily [5]	For 400 mg target dose: • mCYP3Ai: ↓ to 200 mg • sCYP3Ai: ↓ to 100 mg • Posa: ↓ to 50 mg / 70mg^c^ • Ivosidenib: 400 mg ∘ IVO + mCYP3Ai: ↓ 200 mg ∘ IVO + sCYP3Ai: ↓ to 100 mg ∘ IVO + posa: ↓ to 70 mg	• TLS: ensure WBC < 25×10^9^/L before starting venetoclax, follow prescribing information for ramp-up dosing. • Myelosuppression: may require ↓ dose schedules with successive cycles.
**FLT3 inhibitor (type 1 – active against ITD and TKD)**	**Gilteritinib**	120 mg daily [27]	80 mg daily [47]	sCYP3Ai: ↑ levels of gilteritinib. No dose adjustment but monitor closely for AEs.	• ↑ AST/ALT: 14% • Diarrhea/colitis 4% • QTc prolongation: 1%
	**Midostaurin**	50 mg twice daily [113]	50 mg twice daily [51]	sCYP3Ai: ↑ levels of midostaurin but not associated with AEs [114]. ↓ dose not generally recommended, but can ↓ to 50 mg daily with posaconazole in triplets [51]	• Diarrhea/colitis 16% • Rash/desquamation: 14% • ↑ AST/ALT: 13% • Pneumonitis: 8% • QTc prolongation: 6%
**FLT3 inhibitor (type 2 – ITD only)**	**Quizartinib**	53 mg daily^e,b^ [115]	26.5 mg daily^e^ [49]	sCYP3Ai: ↑ levels of quizartinib. 26.5 mg target dose: ↓ to 17.7 mg^e^	• QTc prolongation: 4% • ↑ AST/ALT: 4% • Diarrhea/colitis 2% • Rash/desquamation: 2%
	**Sorafenib**	400 mg twice daily [116]	200-400 mg twice daily [52]	sCYP3Ai: ↑ levels of sorafenib. No dose adjustment but monitor closely for AEs.	• Rash/desquamation: 19% • Hypertension: 10% • Diarrhea/colitis 6% • ↑ AST/ALT: 4%
**Mutant IDH1 inhibitor**	**Ivosidenib**	500 mg daily [61]	500 mg daily [71]	CYP3A inducer and itself a substrate. Will ↓ levels of CYP3A substrates (e.g., VEN). No dose adjustment for sCYP3Ai but monitor closely for AEs.	• DS: 10-20% • QTc prolongation: 10%
	**Olutasidenib**	150 mg twice daily [63]	150 mg twice daily (under investigation)	CYP3A inducer and itself a substrate. May ↓ levels of CYP3A substrates; interaction with VEN is being investigated. No dose adjustment for sCYP3Ai but monitor closely for AEs.	• ↑ AST/ALT: 12% • DS: 10-20%
**Mutant IDH2 inhibitor**	**Enasidenib**	100 mg daily [64]	100 mg daily [71]	CYP1A2 inhibitor. May ↑ levels of CYP1A2 substrates (e.g., caffeine, naproxen).	• DS: 10-20% • ↑ bilirubin: 13% (typically indirect/unconjugated)
**Menin inhibitor**	**Revumenib**	270 mg twice daily^e,d^ [90]	220-270 mg twice daily^e^ [97, 99]	sCYP3Ai: ↑ levels of revumenib. For the following target doses: • 270 mg: ↓ to 160 mg^e^ • 220 mg: ↓ to 110 mg^e^	• DS: 16% • QTc prolongation: 14%
	**Ziftomenib**	600 mg daily [92]	200-600 mg daily (dose-finding ongoing) [100]	No significant interaction with sCYP3Ai.	• DS: 14% • QTc prolongation: 1% grade 1-2, no grade ≥3
	**Bleximenib**^f^	100 mg twice daily [95]	50-100 mg twice daily [117]	Not defined yet.	• DS: 7% • QTc prolongation: 1%
	**Enzomenib**^f^	140-300 mg twice daily (RP2D TBD) [94]	TBD	No significant interaction with sCYP3Ai.	• DS: 12% (all grades) • QTc prolongation: 5% grade 1-2, no grade ≥3

*AE* adverse events, *LIT* low-intensity therapy, *IVO* ivosidenib, *posa* posaconazole, *TLS* tumor lysis syndrome, *WBC* white blood cell count, *mCYP3Ai* moderate CYP3A inhibitor, *sCYP3Ai* strong CYP3A inhibitor, *DS* differentiation syndrome, *VEN* venetoclax, *TBD* to be determined.

^a^This is not an exhaustive list, but rather specific AEs of clinical concern, intended to guide clinician choice if ≥1 agent of a particular class is available. Please consult prescribing information for full AE data.

^b^Data based on early studies in AML. Venetoclax and quizartinib monotherapy are not recommended or FDA approved for AML.

^c^70 mg is the FDA-approved dose in combination with posaconazole.

^d^Follow prescribing guidelines for weight-based dosing if patient weighs <40 kg.

^e^Doses are presented to reflect commercially available tablet sizes. Note that clinical trials have used investigational capsules with slightly different doses (e.g., revumenib 276 mg or quizartinib 30 mg).

^f^Not yet FDA-approved.

Similarly, quizartinib should also be reduced to 30 mg in triplet combinations. The first two patients treated at a dose of 40 mg in the DAC + VEN + QUIZ triplet study developed hematologic dose-limiting toxicities [49]. It should be noted that doses of quizartinib are 60 mg for monotherapy or 40 mg with IC [30, 31], and that quizartinib is commercially available in different dosage forms to those studied in clinical trials. For ease of reference, commercial doses are presented in Table 1, while the manuscript text presents investigational doses to avoid confusion when referring back to the primary literature.

**Fig. 3:**
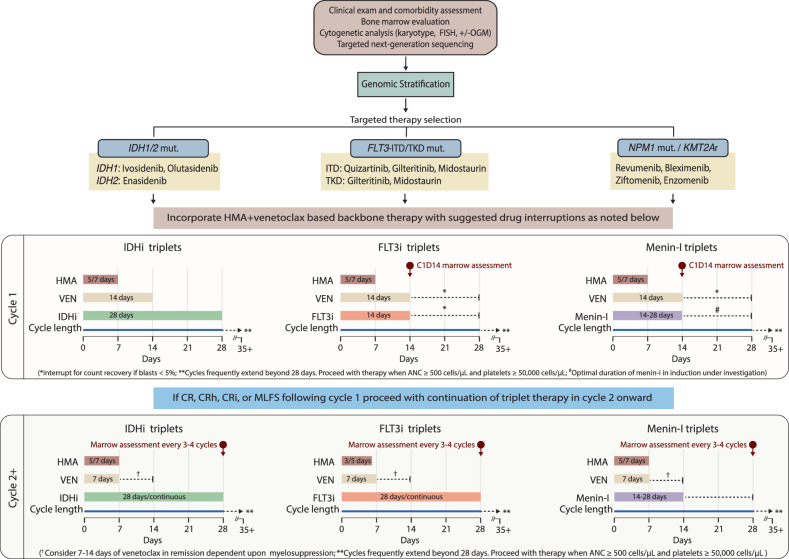
Recommended schedules of triplet combinations. Suggested durations and cycle lengths of triplets are outlined in this figure. Where indicated by dashed lines (e.g., venetoclax and FLT3i duration in cycle 1), duration of agents should be adjusted based on day 14 bone marrow result or cytopenias in previous cycles, respectively.

Dosing of triplet regimens during induction for ND *FLT3*^mut^ AML typically follows the following schedule: AZA 75 mg/m^2^ for 7 days or DAC 20 mg/m^2^ for 5 days, VEN 400 mg for 14 days and gilteritinib 80 mg or quizartinib 30 mg for 14–28 days (Fig. 3). A cycle 1 day 14 (C1D14) bone marrow (BM) biopsy has been used for response-adapted dosing schedules of VEN and FLT3i [49]. Stopping VEN and FLT3i after initial C1D14 response has improved time to ANC > 0.5 × 10^9^/L and platelets >50 × 10^9^/L to 37 days and 27 days, respectively [47, 49]. GCSF is routinely recommended if the C1D14 BM is hypoplastic with <5% blasts, to aid count recovery. Although C1D14 BM should be performed, 95% of responders are hypoplastic at this timepoint [49]. Thus, if C1D14 BM assessment is not feasible, empiric cessation of VEN and gilteritinib is reasonable.

Once remission is attained, further adjustments to dosing schedule in continuation are frequently necessary to minimize myelosuppression. Cycle lengths should be adjusted to allow for adequate hematologic recovery before starting the next cycle. The median post-induction cycle length for FLT3 triplets is 42–49 days [47, 49]. Typical dosing schedules in continuation are AZA 75 mg/m^2^ for 5–7 days or DAC 20 mg/m^2^ for 3–5 days, VEN 400 mg for 7 days, and gilteritinib 80 mg or quizartinib 30 mg continuously.

### Major drug interactions and specific adverse effects

Quizartinib is a substrate of CYP3A; dose reductions to 20 mg are required when concurrent strong CYP3A inhibitors are used, as the risk of QTc prolongation is dose-dependent [30]. While CYP3A inhibitors can increase levels of gilteritinib and midostaurin, dose adjustments for concurrent administration of CYP3A inhibitors are not routinely required; patients should instead be monitored closely for adverse effects with doses adjusted if any are observed.

Grade ≥3 adverse effects of particular concern include QTc prolongation, elevated transaminases, diarrhea/colitis, pneumonitis, rash/desquamation and hypertension, which occur with slightly different frequencies (Table 1) [26, 30, 54]. Differentiation syndrome (DS) can occur in 3–5% of patients treated with FLT3i, and should always be considered in patients with pulmonary infiltrates, hypoxia, weight gain and/or fever [55].

### Anticipated FLT3 triplet studies

Several large studies are in progress to confirm the role of FLT3i triplets. The VICEROY study (NCT05520567) is a multicenter, open-label, randomized, phase 1/2, dose-ranging/expansion study which is evaluating efficacy, safety and optimal dosing of VEN (200 mg versus 400 mg) in the triplet combination of AZA + VEN + GILT. The design of this trial specifies that both VEN and gilteritinib should be held if C1D14 BM showed <5% blasts or <5% cellularity. Early results are expected to be presented at the end of 2025. Importantly, the ongoing MyeloMATCH MM10A-EA02 trial (NCT06317649) is comparing AZA + VEN + GILT in concurrent and sequential dosing schedules to AZA + VEN in a prospective, randomized fashion, with a primary endpoint of MRD-negative CR (sensitivity, 10^−3^) after 4 cycles [56]. A randomized comparison of a FLT3i triplet against IC + FLT3i in older patients fit for intensive chemotherapy is also planned.

## Mutant IDH1/2 inhibitors

Mutations in isocitrate dehydrogenase 1 and 2 (*IDH1*^mut^ and *IDH2*^mut^) occur in 8% and 12% of ND AML, respectively [57, 58]. Patients with *IDH*^mut^ are often older and tend to present with relatively preserved platelet counts [59]. These mutations lead to the accumulation of the oncometabolite 2-hydroxyglutarate (2-HG), which promotes differentiation block and BCL2 dependence [60]. The mutant IDH1 inhibitors ivosidenib [61, 62] and olutasidenib [63] and the mutant IDH2 inhibitor enasidenib [64, 65] relieve this differentiation block and are approved by the FDA as single agents for the treatment of *IDH1*^mut^ and *IDH2*^mut^ AML, respectively. Additionally, ivosidenib in combination with AZA (AZA + IVO) is approved for patients with ND *IDH1*^mut^ AML who are ineligible for IC based on the phase 3 AGILE trial, which showed higher CRc rates (58%) and improved survival (29 months) with AZA + IVO over AZA alone [66, 67].

While AML with *IDH1*^mut^ and *IDH2*^mut^ are typically considered favorable risk according to updated 2024 guidance from the European LeukemiaNet (ELN) due to good outcomes with AZA + IVO and AZA + VEN, respectively [68], relapse is common and long-term survival remains poor [7]. Post-hoc analysis of VIALE-A demonstrated that patients with *IDH2*^mut^ (median OS not reached) have better outcomes compared to those with *IDH1*^mut^ (median OS 15 months), for reasons which are unclear, but which may relate to cytoplasmic versus mitochondrial localization of IDH1 and IDH2, respectively [69, 70]. VEN triggers mitochondrial apoptotic pathways, and has demonstrated synergy with mutant IDH1/2 inhibitors (IDHi), providing the rationale for IDHi triplets [17, 18, 60].

### IDH triplets

Pooled results of two prospective phase 2 trials of AZA + VEN + ivosidenib (AZA + VEN + IVO) for ND *IDH1*^mut^ AML, and decitabine-cedazuridine (DEC-C) + VEN with ivosidenib (DEC-C + VEN + IVO, *IDH1*^mut^) or enasidenib (DEC-C + VEN + ENA, *IDH2*^mut^) were recently reported [71]. Median age was 71 [range, 62–87]; the population included 2 cases with *FLT3*-ITD co-mutation. Results include a CRc rate of 92%, with 87% of responding patients attaining MRD negative status by MFC after a median of 2 cycles (Fig. 2). Responses appear to be durable, with a 2-year cumulative incidence of relapse of 24% and 2-year OS of 70%. With the IDHi triplets, previously described mechanisms of resistance to IDHi monotherapy, such as second-site mutations and isoform switching, have not been observed [72–74]. Of particular interest, 50% of *IDH2*^mut^ and 83% of *IDH1*^mut^ AML relapses occurring in IDH triplet treated patients have been *IDH* wildtype, suggesting effective eradication of the original *IDH*^mut^ AML and evolution or progression of a separate clone.

### Recommended triplet doses and schedules

In both reported IDH triplet studies, VEN is administered on days 1–14 of each treatment cycle, with the IDHi starting on C1D15 of AZA + VEN + IVO and C1D8 of DEC-C + VEN + IVO/ENA and given continuously thereafter. Once the safety and efficacy of this triplet were established, the delayed IDHi start in cycle 1 has remained for practical reasons of IDHi drug acquisition. If accessible, IDHi can start concurrently with the HMA + VEN in cycle 1.

Importantly, IDH triplets appear well tolerated without substantial increased myelosuppression or toxicity compared to HMA + VEN regimens alone. The grade ≥3 thrombocytopenia rate was 2% for ivosidenib and 18% for enasidenib monotherapy [61, 65]. With the triplet, the 60-day mortality was 2%, despite the older population. The median time to ANC > 0.5 × 10^9^/L and platelets >50 × 10^9^/L was 34 days and 20 days, respectively. The median cycle length for IDH triplets was 35 days, 7–14 days shorter than FLT3 triplet cycle length, despite more VEN exposure in continuation cycles [71].

For induction, we suggest the following schedule: AZA 75 mg/m^2^ for 7 days or DEC-C 35 mg/100 mg for 5 days, VEN 400 mg for 14 days and ivosidenib 500 mg daily or enasidenib 100 mg daily for *IDH1*^mut^ and *IDH2*^mut^ AML, respectively. In continuation, depending on tolerability of previous cycles, AZA may be given for 5–7 days, DEC-C for 3–5 days and VEN for 7–14 days (Fig. 3). Reduction in the duration of ivosidenib and enasidenib in continuation cycles is not typically required.

### Major drug interactions and specific adverse effects

Ivosidenib is a CYP3A inducer and hence decreases concentrations of drugs which are CYP3A substrates, such as VEN. A phase 1 study of ivosidenib and VEN found that concurrent administration decreased VEN levels by 45–55% [74]. In the AZA + VEN + IVO and DEC-C + VEN + IVO trials, the recommended phase 2 dose (RP2D) of VEN was 400 mg and 600 mg, respectively, in the absence of CYP3A inhibitors [71]. The RP2D differed between trials, as 600 mg was not evaluated in the earlier AZA + VEN + IVO study, and later data suggested VEN can be safely given at higher doses in combination with ivosidenib. Although dose-limiting toxicities were not reached with 800 mg of VEN, the lower doses were chosen based on comprehensive assessment of efficacy, tolerability and durability of responses [74]. Doses of VEN need to be further adjusted if concurrent CYP3A inhibitors are given (Table 1) [71].

DS occurs in 10–20% of patients treated with single-agent IDHi, with a median time to onset of approximately 20 days [55, 75]. Although most cases are steroid-responsive, fatal events may occur in up to 5% [75]. Importantly, clinical response and occurrence of DS do not always correlate; it is possible to have DS in a non-responding patient [76]. The incidence of DS appears to be lower (5%, grade ≥3, 3%) with the IDH triplets, possibly due to concurrent anti-leukemic agents preventing uncontrolled differentiation [71]. Steroid prophylaxis was not routinely recommended on the IDHi triplet trials, although hydroxyurea for persistent leukocytosis was allowed.

Aside from DS, other significant grade ≥3 adverse events include: QTc prolongation, elevated bilirubin and elevated transaminases (Table 1) [61, 77, 78]. Of note, the raised bilirubin seen with enasidenib is due to inhibition of UGT1A1, and is hence frequently indirect/unconjugated and not clinically significant. It is not typically associated with elevated transaminases or liver dysfunction and generally resolves without treatment discontinuation [77].

### Anticipated IDH triplet studies

Other studies of IDH triplets are in progress but have yet to report outcomes. These include olutasidenib in combination with AZA + VEN (NCT06782542) or DEC-C + VEN (NCT06445959) [79], and LY3410738, a potent dual inhibitor of IDH1/2, with AZA + VEN (NCT04603001). The European HOVON/AMLSG group is now enrolling a randomized phase 3 study of AZA + IVO with VEN or placebo in *IDH1*^mut^ AML (EVOLVE-1, NCT07075016) to definitively answer the question of whether the triplet improves event-free survival (EFS) compared to AZA + IVO alone. For *IDH2*^mut^ AML, a phase 2 MyeloMATCH study is comparing DEC-C + VEN to DEC-C + VEN + ENA, with a primary outcome of MRD-negative CR after 2 cycles of treatment, assessed by MFC (NCT06672146).

## Menin inhibitors

Rearrangements of *lysine methyltransferase 2A (KMT2A)*, located on chromosome 11q23, occur in 5–10% of pediatric and adult acute leukemias [80]. *KMT2A*r confer a poor prognosis with standard therapies, especially in older patients [81]. Mutations in *nucleophosmin 1* (*NPM1*^mut^) are present in up to 30% of ND AML and 12% of RR AML, and result in defective shuttling and cytoplasmic persistence of NPM1 [82–84]. Although considered favorable risk, *NPM1*^mut^ in the setting of mutations in *FLT3*-ITD, *NRAS, KRAS* or *TP53* is associated with disappointing outcomes when treated with HMA + VEN (median OS 10 months) [85]. Both *NPM1*^mut^ and *KMT2Ar* drive leukemogenesis in part through constitutive activation of transcriptional homeobox (*HOX*) genes, which regulate cell proliferation and differentiation [86–88]. Menin is a scaffold protein that interacts with such transcription factors to regulate gene expression [80]. Menin inhibitors are thus active in specific subtypes of acute leukemia (*KMT2A*r, *NPM1*^mut^, *NUP98* rearrangements, etc.) by targeting the transcriptome complex and relieving differentiation arrest [89].

Revumenib is the first menin inhibitor to be FDA approved for RR AML with *KMT2A*r or *NPM1*^mut^. As a single agent, the CR/CRh (complete remission with partial hematologic recovery) rate was 23% in RR *KMT2A*r and *NPM1*^mut^ AML [90, 91]. Ziftomenib, FDA approved for RR *NPM1*^mut^ AML, is associated with a 35% CR rate in patients treated at the RP2D [92, 93]. Ziftomenib is no longer in development for *KMT2A*r as monotherapy, because of a lower response rate and a higher incidence of severe DS [92]. Other menin inhibitors in clinical trials include enzomenib (NCT04988555), bleximenib (NCT04811560), and BN104 (NCT06052813), which appear to have similar single-agent efficacy in phase 1 studies [94–96].

### Menin inhibitor triplets

Triplet combinations with an HMA + VEN backbone are an area of active investigation. Although most data presented have been in RR AML, a BEAT AML substudy recently reported the outcomes of the combination of AZA + VEN with revumenib (AZA + VEN + REV) in ND *KMT2A*r or *NPM1*^mut^ AML [97]. This phase 1 study evaluated revumenib at two dose levels—226 mg or 276 mg (without strong CYP3A inhibitors) and 113 mg or 163 mg (with strong CYP3A inhibitors), with continuous VEN on days 1–28. Both doses of revumenib were found to be safe. The study enrolled 43 patients with a median age of 70 (40% aged ≥75), with a CRc rate of 81% (80% in *NPM1*^mut^ and 89% in *KMT2A*r) and 100% MRD undetectable status by MFC (31% by *NPM1* NGS, Fig. 2) [97]. Of the 34 (79%) patients with *NPM1*^mut^ AML, 17 (50%) were favorable and 17 (50%) intermediate risk by ELN 2024, with a median OS of 15.5 months. There were 5 (12%) early deaths prior to disease assessment, including 2 patients who transitioned to hospice. Despite deep remissions and minimal relapses, the early rates of discontinuation and mortality highlight the importance of dose optimization for safe administration of this regimen.

Early results of the triplet combination of AZA, VEN and bleximenib (AZA + VEN + BLEXI, NCT05453903) have been presented [98]. This ongoing phase 1b study evaluated varying doses of bleximenib in ND (30, 50 and 100 mg twice daily) and RR (15, 30, 50, 80, 100 and 150 mg twice daily) *KMT2A*r or *NPM1*^mut^ AML. Efficacy data were reported from the 50 and 100 mg ND cohorts (50 mg *n* = 13, 100 mg *n* = 20), with CRc in 75% of ND *NPM1*^mut^ and *KMT2A*r AML. Responses appeared better with 100 mg (ND overall response rate [ORR] 90%) compared with 50 mg (ND ORR 76%) of bleximenib; no further improvement in efficacy was reported in the 150 mg RR cohort. Of the 16 patients in the 100 mg cohort with ND *NPM1*^mut^ AML, 12 (75%) remained on treatment at the time of presentation (median 3 [range, 1–3] cycles per patient), 1 relapsed, 2 discontinued at treating physician discretion and 1 discontinued for unspecified reasons.

In RR AML, revumenib (NCT05360160) [99], bleximenib (NCT05453903) [98], ziftomenib (NCT05735184) [100] and enzomenib (NCT04988555) are all being evaluated in combination with HMA + VEN, with CRc rates between 30% and 50%, despite enrolling heavily pre-treated and high-risk patients [89].

### Recommended triplet doses and schedules

In the BEAT AML substudy, AZA + VEN + REV was administered as follows: AZA 75 mg/m^2^ for 7 days, VEN 400 mg daily for 28 days and revumenib 226 mg (dose level [DL] 1) or 276 mg (DL2) twice daily for 28 days [97]. In the setting of grade 4 neutropenia or thrombocytopenia lasting >14 days after cycle completion, the first required dose adjustment was a reduction in revumenib duration to 21 days, followed only by a second reduction in VEN duration to 21 days if grade 4 cytopenias were still persistent in subsequent cycles despite the revumenib dose adjustment. The protocol was amended in September 2024 to reduce the number of days of VEN in the continuation phase to 14 days (revumenib remained at 28 days), with subsequent dose reductions based on persistent grade 4 cytopenias lasting >14 days after completion of a continuation-phase cycle. Despite these dose adjustments, the median cycle length in the continuation phase was 42 days, with 46% of cycles delayed by ≥2 weeks.

In the phase 2 study of the all-oral combination of revumenib, DEC-C and VEN (SAVE, NCT05360160), DEC-C was administered at 35 mg/100 mg for 5 days, VEN at 400 mg for 14 days, and revumenib at 276 mg (without strong CYP3A inhibitors) or 163 mg (with strong CYP3A inhibitors) twice daily for 28 days [99]. All patients underwent BM assessment on C1D14, with revumenib held after D21 if BM blasts were <5%. There was no early (<60 days) mortality reported in the RR cohort; the ND cohort has not been reported yet.

Given the myelosuppression seen with menin triplets, we recommend the following dose schedules based on target doses, with careful dose adjustments given drug interactions with CYP3A inhibitors (Table 1). Induction: AZA 75 mg/m^2^ for 7 days or DEC-C 35 mg/100 mg for 5 days, VEN 400 mg for 14 days and revumenib 276 mg twice daily for 21–28 days (depending on result of C1D14 BM). Frontline menin triplet trials have investigated revumenib from days 21-28 in induction. However, the early mortality and discontinuation rates suggest that shorter revumenib durations may be adequate if the C1D14 BM is clear. For continuation, we suggest: AZA 75 mg/m^2^ for 5–7 days or DEC-C 35 mg/100 mg for 3-5 days, VEN 400 mg for 7–14 days and revumenib 276 mg twice daily for 14–28 days (Fig. 3). Given the high risk of CNS involvement/relapse in *KMT2A*r, and the propensity for extramedullary disease in *NPM1*^mut^ or monocytic AML, coupled with the lack of CNS penetration of menin inhibitors, we recommend 2-4 doses of intrathecal cytarabine for CNS prophylaxis. Combination dose-determination is ongoing for ziftomenib, bleximenib and enzomenib.

### Major drug interactions and specific adverse effects

Revumenib is a major substrate of CYP3A. Levels are increased when co-administered with strong inhibitors of CYP3A and hence dose reduction is required. Target doses of 276 mg and 226 mg should be reduced to 163 mg and 113 mg, respectively, when given with strong CYP3A inhibitors such as posaconazole or voriconazole. Ziftomenib and enzomenib levels do not appear to be significantly altered with exposure to tri-azoles [92, 94].

Revumenib can significantly prolong the QTc. Co-administration with other QT-prolonging drugs should be avoided where possible. Grade ≥3 QTc prolongation was seen in 14% of patients treated with revumenib, but is much less common with blexmenib, ziftomenib and enzomenib (Table 1) [90–92, 94, 95].

**Fig. 4:**
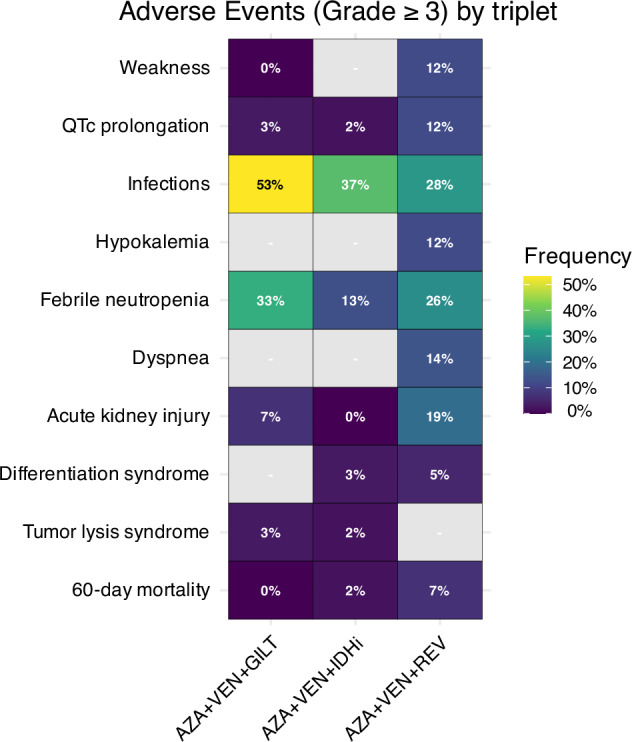
Adverse events. Grade ≥3 non-hematologic adverse events for reported triplet regimens [47, 71, 97].

As with IDHi, DS is a concern with menin inhibitors. Grade ≥3 DS occurred in 16% of patients treated with revumenib [90], 14% with ziftomenib, 7% with bleximenib [95] and 12% (all grades) with enzomenib [94]. Importantly, in some cases, severe DS may manifest with signs and symptoms resembling cytokine release syndrome [89]. As DS can be fatal, early recognition and prompt initiation of corticosteroids and/or cytoreduction are critical [55]. As with IDHi, triplet therapy seems to reduce, but not abrogate, the risk of DS. Although overall rates (19%) of DS were similar on AZA + VEN + REV, grade ≥3 events were reported in only 4% of patients treated with AZA + VEN + BLEXI and 5% with AZA + VEN + REV [97, 98]. An overview of grade ≥3 non-hematologic adverse events by triplet regimen is shown in Fig. 4.

### Anticipated menin triplet studies

The first reports of the ND cohort of SAVE and the combination of AZA, VEN and enzomenib (NCT04988555) are expected to be presented at the 2025 American Society of Hematology Annual Meeting. In addition, large phase 3 randomized clinical trials of AZA + VEN with placebo or revumenib (HOVON group, NCT06652438), ziftomenib (KOMET-017-NIC, NCT07007312), or bleximenib (cAMeLot-2, NCT06852222) will hopefully definitively address the role of menin inhibitor triplets in the treatment of *NPM1*^mut^ and *KMT2A*r AML.

## Future considerations and conclusion

AML is a heterogeneous disease with complex clonal ontogeny. Various triplet therapies aim to build on the foundation of HMA + VEN doublets to target leukemic clones and pre-empt primary and secondary mechanisms of resistance and relapse. The deep responses seen with triplet therapies may also enable more older adults to transition to curative allogeneic stem cell transplant, while oral formulations of decitabine and azacitidine with cedazuridine will hopefully soon enable general adoption of total oral therapy for AML [101].

Future studies must explore how best to sequence and incorporate available agents to potentially cure AML while avoiding excessive toxicity. Innovative and adaptive trial designs will be required to meet this need. Current concepts include quadruplet therapy, or alternating sequential therapies, to allow different agents to be administered separated by time, allowing for recovery between exposures. These approaches have had success in AML (cladribine with LDAC + VEN alternating with AZA + VEN) [102] and acute lymphoblastic leukemia (hyperfractionated cyclophosphamide, vincristine, doxorubicin and dexamethasone [HyperCVAD] alternating with methotrexate and cytarabine, with or without incorporation of tyrosine kinase inhibitors, VEN, nelarabine and/or asparaginase) [103–105], amongst others. Triplet therapies including a mutation agnostic agent (e.g., those incorporating a CD123-directed therapy [106] or a purine analog [102]) may be of interest in challenging subsets such as *TP53* mutated AML. Trials exploring how to sequence available therapies are also of interest and are ongoing. For example, the I-DATA trial (NCT05401097) is a randomized phase 2 study which aims to compare the overall treatment failure rate at 24 months in patients with ND *IDH1*^mut^ or *IDH2*^mut^ AML. Patients will be randomized to receive a frontline doublet of either AZA + IVO (*IDH1*^mut^)/AZA + ENA (*IDH2*^mut^) or AZA + VEN, followed by treatment with the other doublet at relapse [107].

While continued therapy appears to be necessary to maintain remission, the question of whether all three agents are required indefinitely remains unanswered. It is already apparent that incorporation of novel therapies is altering the cytomolecular landscape and trajectory of relapsed AML. Patients with AML harboring founder mutations such as *NPM1* can lose these mutations at relapse after VEN-based therapies [108], patients treated with FLT3i and IDHi triplets similarly appear more likely to relapse without persistence of these mutations [50, 51, 71], and more intense cytarabine-based backbones appear to reduce the frequency of emergent *FLT3*-ITD at relapse [109, 110]. Long-term continuation phases of these regimens may eventually omit one or more drugs, allowing continued therapy with improved quality of life and reduced cost for patients in deep and ongoing remissions. Such approaches also present an opportunity to conduct research into financial toxicity of combination therapy and estimates of quality-adjusted life years gained from such therapies.

In conclusion, we provide a practical framework for the use of triplet regimens in AML. These recommendations are not intended to be prescriptive or exhaustive, but rather to highlight the complexity of drug delivery and the continuous refinements to dosing schedules which are required to minimize toxicity while allowing the efficacy of triplets to be harnessed. Ongoing phase 3 randomized controlled trials will hopefully provide definitive answers to the role of triplets in the treatment of AML, and are necessary given prior mutation-agnostic triplets which had shown early promise did not met their primary endpoint in confirmatory Phase 3 studies [111]. Furthermore, new studies addressing altered relapse dynamics and how to best incorporate the increasing number of active agents in AML should be undertaken.
